# Corrosion protection performance of silicon-based coatings on carbon steel in NaCl solution: a theoretical and experimental assessment of the effect of plasma-enhanced chemical vapor deposition pretreatment†

**DOI:** 10.1039/d1ra08848c

**Published:** 2022-05-25

**Authors:** Amel Delimi, Hana Ferkous, Manawwer Alam, Souad Djellali, Amel Sedik, Kahlouche Abdesalem, Chérifa Boulechfar, Amina Belakhdar, Krishna Kumar Yadav, Marina M. S. Cabral-Pinto, Byong-Hun Jeon, Yacine Benguerba

**Affiliations:** Laboratoire de Génie mécanique et Matériaux, Faculté de Technologie, Université de 20 août 1955 de Skikda Skikda 21000 Algeria; Département de Technologie, Université de 20 août 1955 de Skikda Skikda 21000 Algeria; Department of Chemistry, College of Science, King Saud University P.O. Box 2455 Riyadh 11451 Saudi Arabia; Laboratoire de Physico-Chimie des Hauts Polymères (LPCHP), Faculty of Technology, University Ferhat Abbas Setif1 19000 Setif Algeria; Scientific and Technical Research Center in Physico-chemical Analysis BP 384, Bou-Ismail industrial zone, RP 42004 Tipaza Algeria; Nanomaterials, corrosion and surface treatment laboratory (LNMCT), BP 12, Badji Mokhtar University 23000 Annaba Algeria; CRTI Research Centre in Industrial Technologies – CRTI P.O. Box 64 Cheraga 16014 Algiers Algeria; LaboratoireMatériaux et SystèmesElectroniques, Universityof BordjBouArreridj 34000 Algeria; Faculty of Science and Technology, Madhyanchal Professional University Ratibad Bhopal 462044 India; Geobiotec Research Centre, Department of Geoscience, University of Aveiro 3810-193 Aveiro Portugal; Department of Earth Resources and Environmental Engineering, Hanyang University Seoul 04763 Republic of Korea bhjeon@hanyang.ac.kr; Department of Process Engineering, Faculty of technology, Ferhat Abbas Setif1 University Setif Algeria yacinebenguerba@univ-setif.dz

## Abstract

Using a plasma-assisted chemical vapor deposition (PACVD) process, carbon steel samples were coated with an organosilicon layer less than 2.5 microns thick. Ellipsometry, Fourier transform infrared (FTIR) spectroscopy, contact angle, scanning electron microscopy (SEM), and atomic force microscopy (AFM) were used to analyze the films. Additionally, gravimetric experiments were used to determine the electrochemical properties of the organosilicon coatings. Organosilicon-coated carbon steel specimens demonstrated significantly enhanced resistance to corrosive conditions, such as 3% aqueous sodium chloride solutions. The surface preparation method has a considerable influence on the morphological and electrochemical properties of the steel. Argon pretreatment significantly enhances the corrosion resistance of organosilicon-coated steel. Gravimetric research demonstrated that pretreatment with argon plasma resulted in less weight loss and corrosion than pretreatment with nitrogen plasma. The link between quantum computing and experimental data using density functional theory (DFT) and molecular dynamics (MD) was used.

## Abstract 2

Using a plasma-assisted chemical vapor deposition (PACVD) process, carbon steel samples were coated with an organosilicon layer less than 2.5 microns thick.

## Graphical abstract

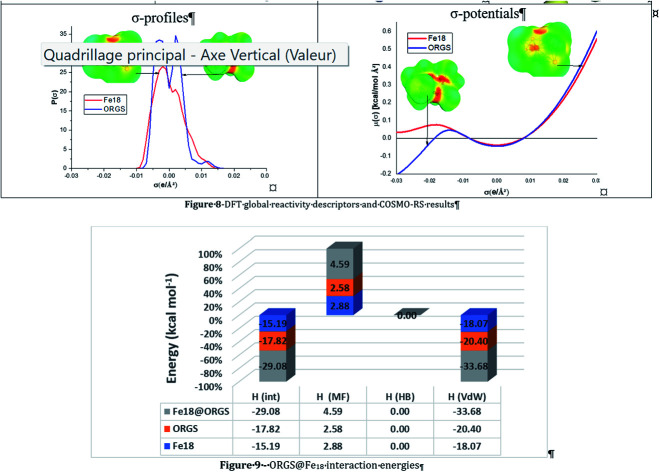

## 1. Introduction

Metals that rust can't be utilized in key sectors like aircraft and transportation because of the natural process known as corrosion. Corrosion is a common problem for metallic alloys in industrial applications because of the conditions in which they are used. Applying a protective coating or paint is highly recommended as the most cost-effective and practical strategy to prevent corrosion. Even though coatings and paints are relatively thin, they may be quickly applied over broad areas and provide protection. In most cases, the cost of materials and labor is less than the value of what is being protected. Corrosion-resistant coating and paint technology have advanced consistently over the past 150 years, resulting in a diverse spectrum of solutions for preserving a variety of substrates in a variety of situations. The coatings industry, a mature field, is continually developing novel substrates and application procedures to meet new needs. Coating technology has advanced dramatically during the previous three decades to respond to environmental concerns. As a result, new coating systems and application procedures are being developed.

Organic coatings and anticorrosive paint mixes are a common means of preventing corrosion on metal surfaces. The hydrophobic nature can be studied with a dynamic contact angle. This quantitative method shows the increase in hydrophobicity and the decrease in the wettability when organic coatings include fluorinated groups.^5^ Corrosion mitigation techniques include covering metals with protective coatings that prevent corrosion ions and molecules from accessing their surfaces.

Numerous organic coatings may be modified to improve their surface and bulk properties. Numerous modern coatings are formulated to produce hydrophobic/superhydrophobic surfaces, reliable substrate/coatings interface adhesion, and long-term corrosion protection.^1^

Amorphous films such as hydrogenated silicon carbide (a-SiC_*x*_ : H), silicon nitride (a-SiN_*x*_ : H), and silicon carbonitride (a-SiC_*x*_N_*y*_ : H) were used as protective coatings on stainless steel.^1–3^ Corrosion tests performed in 1% sodium chloride solution revealed that the coated samples possessed substantially better corrosion resistance than the bare substrate.

Chromate conversion coatings have gained popularity due to their application ease.^2^ These procedures have significant environmental consequences because of the need for potentially hazardous reactants and the generation of toxic waste. As a result, other technologies have been examined. PACVD (plasma-assisted chemical vapor deposition) is used in various sectors, including semiconductor production.^3,4^ Different organosilicon (ORGS) precursors may be used to fabricate thin films with regulated physicochemical properties. The features of the growing film may be precisely controlled by modifying parameters such as gas flow rates, gas ratios, and even the kind of plasma employed.^5^ Plasma Assisted Chemical Vapor Deposition (PACVD) is a technique of choice for developing amorphous materials.^4^ This character is generally associated with a low electrical conductivity of the layers, which avoids any galvanic coupling with the metal substrate, which is thus protected. This technique results in adherent and dense films with a relatively high deposition rate (μm h^−1^).

The substrate may be prepared immediately before beginning the coating process to improve adhesion further. The researchers believe this, along with the process's low impact on the environment, makes it a viable option for improving metal corrosion resistance. PACVD-grown amorphous ORGS films exhibit a high electrical resistivity (10^9^ to 10^15^ cm), characteristic of amorphous ORGS films.^6,7^ In other words, the conditions surrounding the formation of deposits may significantly impact their quality. To boost the barrier properties for corrosion applications, the number of Si–O bonds must be increased, and the hydrogen concentration in the coating decreased.^8^

Corrosion inhibition was investigated using ORGS coatings, which improved the material's corrosion protection properties and can be synthesized using a straightforward PACVD process.

Furthermore, Density Functional Theory (DFT) is an effective technique for analyzing the corrosion inhibition of many ORGS, with findings correlating with actual data.^9^ Additionally, DFT simulations were used to deduce the mechanism of ORGS adsorption on the carbon steel surface. This study focuses on the corrosion behavior of carbon steel that has been insulated and prevented from rusting by thin ORGS coatings. After 100 hours of immersion in a solution containing 3% sodium chloride, the corrosion resistance of the interface materials was determined.

## 2. Materials and methods

### 2.1. Materials

Arcelor in France provided the carbon steel substrates, which contain 0.37% C, 0.23% Si, 0.68% Mn, 0.5% Cr, 0.16% Cu, 0.059% Ni, 0.016% S, 0.011% Ti and 97.9% Fe. The NaCl was provided by Aldrich.

### 2.2. Surface coating

#### 2.2.1 SiO_*x*_-like thin films preparation

The carbon steel samples were degreased with Milli-Q water and thoroughly cleaned in an ultrasonic bath with 2-propanol and acetone at ambient temperature before drying under a nitrogen stream. Next, the steel substrates had to be heated to 300 °C at a pressure of only 0.005 Torr for an hour in the plasma chamber. The SiO_*x*_ layers were deposited using PACVD in a Plasmalab 800Plus (Oxford Instruments, UK). The following growth conditions were used: a substrate temperature of 300 °C, a gas mixture of N_2_O and SiH_4_, a gas flow rate of N_2_O: 260 SCCM and SiH_4_ (4% N_2_): 700 SCCM for a 1 Torr total pressure, and a ten Watts power at a 13.56 MHz frequency. As a result, silica films with a refractive index of 1.54 were deposited at 414 Å min^−1^. The silica film thickness may be changed while the deposition time was kept constant at ten minutes.

The following settings were used before the deposit: (i) Ar Pre-treatment: flow rate = 500 SCCM, time = 5 min; N_2_ pre-treatment: flow rate = 2500 SCCM, time = 5 min.

### 2.3. Surface characterization

#### 2.3.1 Micro-Raman analysis

A Jobin-Yvon T64000 spectrometer obtained micro-Raman spectra at 514.5 nm using a 10× objective and a CCD camera. The spectral range studied was 10–1400 cm^−1^, with a precision of 0.5 cm^−1^.

#### 2.3.2 Scanning electron microscopy (SEM)

A thermal field emission emitter and three separate detectors (ESB detector with filter grid, Everhart-Thornley secondary electron detector, and high-efficiency In-lens SE detector) were used to take the SEM images.

#### 2.3.3 Electron microprobe analysis (EMPA)

Electron microprobe investigation was used to analyze the surface morphology of the coatings and corroded interfaces. The samples were sputter-coated using a Bal-tec SCD005 supper coater (France), and a Cameca SX100 electron probe microanalyzer was used to analyze the elements. Back-scattered electron images were taken at a voltage of 15 kV and a current of 20 nA.

#### 2.3.4 Contact angle measurements

The contact angles between water and metal surfaces were determined using a computer-controlled goniometer (DIGIDROP by GBX). The accuracy is 2°. All measurements, unless otherwise noted, were taken at room temperature.

#### 2.3.5 Ellipsometry

The visible range spectroscopic ellipsometry data were acquired using a UVISEL Jobin Yvon Horiba Spectroscopic Ellipsometer coupled with the data processing software DeltaPsi 2. The system obtained a spectrum extending from 2 to 4.5 eV (300–750 nm) with intervals of 0.05 eV (or 7.5 nm). The angle of incidence was adjusted to 70, and the compensator was set to 45. Regression analysis was used to fit the data to a film-on-substrate model defined by the film thicknesses and the two complicated refractive indices.

#### 2.3.6 Electrochemical measurements

Electrochemical measurements were performed utilizing a standard three-electrode configuration consisting of a saturated calomel reference electrode (SCE), a platinum grid serving as a counter electrode, and a Potentiostat/Galvanostat “Solartron 1255B Frequency Response” analyzer. The working electrode was made from a cylindrical XC38 steel rod and insulated with polytetrafluoroethylene (PTFE) tape to create a surface area of *A* = 7.5 cm^2^. At 30 °C, aerated 3% NaCl was used to conduct tests on uncoated and coated carbon steel samples. The applied voltage was found at 0.5 mV s^−1^ scan rates.^10,11^ For comparative purposes, it was required to record the cathodic and anodic fields separately. Electrochemical impedance spectroscopy (EIS) was performed spanning eight frequency decades at an open circuit potential (OCP) of 2 mHz to 100 kHz utilizing a Solartron SI 1287 electrochemical interface. The impedance measurements were simulated using ZView2.

#### 2.3.7 Gravimetric experiments

Gravimetric measurements in a double-glass cell were made using a temperature-controlled cooling condenser. The solution contained 100 milliliters. The specimens used were rectangular (2.3 cm long, 1 cm wide, 0.1 cm thick).

### 2.4. Theoretical study

#### 2.4.1 Quantum chemical calculations

The ORGS and Fe_18_ cluster structures were optimized using the DFT-B3LYB functional and the Turbomole package's TZVP basis set.^12^ The Conductor-like Screening Model for Real Solutions (COSMO-RS) was computed using the COSMOTherm program.^12–14^

From the ionization (*I*) and electron affinity (*A*) ground states, it can deduce the hardness (*η*) and chemical potential (*μ*) or electronegativity (*χ*) equations, respectively.1
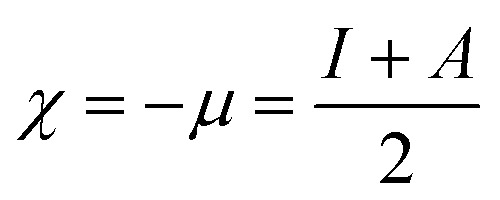
2
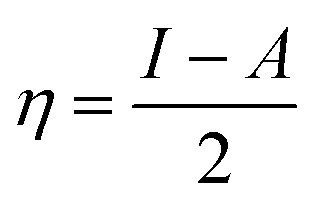

3 *I* = −*E*_HOMO_

4 *A* = −*E*_LUMO_

*E*_HOMO_ and *E*_LUMO_ represent the energy of HOMO and LUMO orbitals, respectively.

The electronegativity and hardness values determine an ion, atom, or molecule's electrophilicity index (*ω*).^14^5
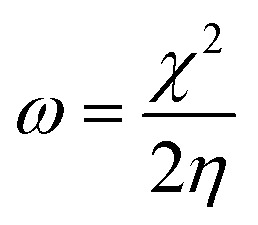


The percentage of electrons transported from the ORGS molecule to the carbon steel sample's surface is computed as follows:^14^

6 Δ*N* = (*χ*_Fe_ − *χ*_ORGS_)/2(*η*_ORGS_ + *η*_Fe_)

#### 2.4.2 Bader's analysis (AIM)

The atoms in a molecule (AIM) theory might help us better understand intermolecular interactions. The kind and strength of molecular bonding connections may be analyzed using molecular electron densities *ρ*(*r*). ∇^2^*ρ*(*r*) may be used to identify the kind of chemical bonding present at the bond critical point (BCP), where *ρ*(*r*) is either minimum or maximal.^15,16^

*V*(*r*) and *G*(*r*), the capacity to aggregate electrons and dilute them *via* electronic mobility, are in rivalry.^17^ As a consequence, *H*(*r*) = *V*(*r*) + *G*(*r*) may have a negative or positive total energy density.

Furthermore, positive, ∇^2^*ρ*(*r*) and *H* values indicate electrostatic contact, while negative values suggest a covalent bond. A partly covalent bond^18^ is defined as a positive ∇^2^*ρ*(*r*) and a negative *H* value. The categorization of interactions is therefore based on the values of that indicator, according to these many possibilities: (I) pure shell interaction, |*V*|/*G* < 1; (II) shell interaction, 1 < |*V*|/*G* < 2; (III) layer sharing interaction, |*V*|/*G* > 2. The first two forms of interactions are based on hydrogen bonds, whereas the third type is covalent bonds.

ADF software^19,20^ was used to calculate AIM. For molecular structure optimization, the DFT-B3LYP was utilized using the def-TZVP basis set.^20,21^

## 3. Results and discussion

### 3.1. ORGS film preparation and characterization

#### 3.1.1 SiO_*x*_-like films deposition

The silica coatings examined in this study were produced on carbon steel substrates through plasma-assisted chemical vapor deposition (PACVD) at 300 °C.^22,23^ The gas carriers utilized were silane (SiH_4_) in N_2_ at a concentration of 3% and nitrous oxide (N_2_O) at a concentration of 3%, as previously described for the deposition of thin SiO_*x*_ coatings onto gold substrates. Indeed, it was assumed that the presence of N_2_O during deposition was responsible for the oxide film's strong adherence to the noble metal interface, which did not delaminate even after piranha treatment at 80 °C.^22^ Under these circumstances, homogeneously deposited silica films with a refractive index of *n* = 1.48 and a deposition rate of 414 Å min^−1^ were obtained.

The deposition duration determined the deposited layer's thickness, which was 20 minutes. Profilometric data were used to determine the ORGS layer's thickness, which came out to 2.5 ± 0.1 μm. The interfaces were treated with nitrogen and argon plasmas before the coating was deposited. The final wetting parameters of the coated sample were significantly influenced by the kind of carbon steel surface pretreatment. The static contact angle of polished uncoated carbon steel is 89 ± 2°. With and without any specific preparation or nitrogen plasma treatment, a 2.5-micron thick organosilicon layer significantly increased the water contact angle to 95 ± 2° and 98 ± 2°. However, when pretreated with argon, hydrophobic water contact angles were determined to be 117 ± 2°. This result indicates that the argon-treated film minimizes the contact area between the solution and the solid surface of the metal, thus reducing corrosion.

The wetting parameters of the argon-pretreated contact are very stable throughout time. Indeed, after 20 days, no change in the wetting properties was observed.

**Fig. 1:**
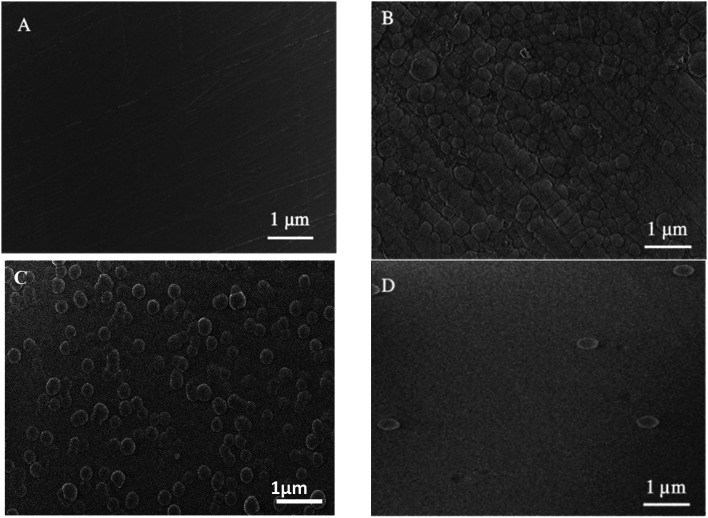
Surface microstructure of XC38 with various surface pretreatments coated with ORGS: (A) XC38 polished, (B) no pretreat. (C) N_2_ pretreat. (D) Ar pretreat.

**Fig. 2:**
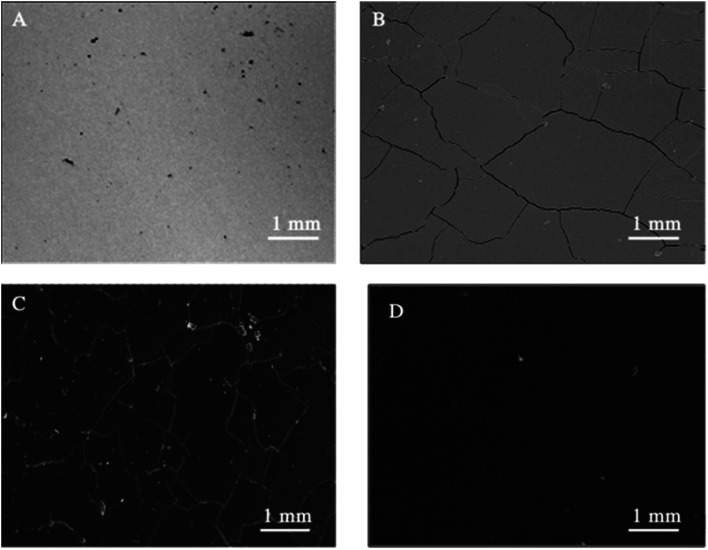
Microprobe characterization of XC38 with various pretreats coated with ORGS: (A) XC38 polished. (B) no pretreat. (C) N_2_ pretreat. (D) Ar pretreat.

SEM (Fig. 1) and electron microprobe (Fig. 2) investigations examined the interface morphology. The argon pretreatment of carbon steel surfaces before the deposit of the ORGS layer results in an ultra-thin polymer coating with refined grains as contrasted to carbon steel surfaces that have not had nitrogen pretreated or have not been treated at all. The findings of the SEM may be verified using images of back-scattered electrons taken with an electron microprobe. Argon pretreatment results in a smooth surface with minimal defects, while untreated and nitrogen plasma pretreated samples show fractures due to the lack of treatment. Corrosion-resistant coatings may be less effective if exposed to moisture. Although imperfections like holes and cracks make it easier for solutions to penetrate, the carbon steel surface underneath is targeted.

### 3.2. Raman characterization

**Fig. 3:**
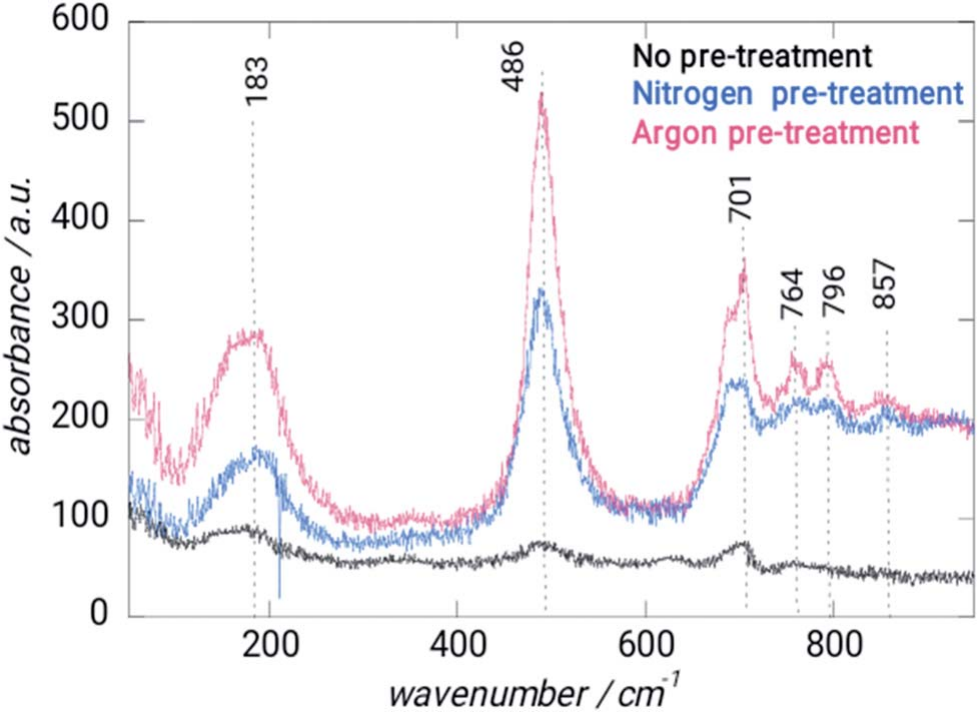
Raman spectrum of XC38 steel without and with different treatments.

Spectroscopic data has been gathered using Raman spectroscopy (Fig. 3). Because of the deposit's pseudo amorphous structure, the band at 183 cm^−1^ is ascribed to the crystal lattice mode, while the 486 and 701 cm^−1^ bands are attributed to the semiconductor Si–O–Si.^22–24^

Between 750 and 860 cm^−1^, the CH deformation modes may be detected. EDS analysis verified the coating's amorphous nature (Table 1). Pretreatment with argon results in a bit of reduction in the Si/C ratio, but the Si/O composition remains mostly constant. The Ar–Ar plasma treatment of ORGS resulted in a smooth and homogenous surface. Because of the enhanced energy transfer from the Ar plasma to the ORGS surface, the formerly loose nanostructure has become more compact as the treatment time with Ar plasma has risen, which explains why the carbon content is growing as a percentage.

**Table 1:** EDS parameters for plasma coated steel

Sample	Atomic percentage/%				
	Si	O	C	Si/O	Si/C
ORGS coated steel (no pretreatment)	31.1	51.6	17.3	0.6	1.8
ORGS coated steel (N_2_ pretreatment)	30.8	50.6	18.6	0.6	1.7
ORGS coated steel (Ar pretreatment)	28.9	53.1	18.0	0.5	1.6

### 3.3. Investigation of the corrosion protection efficiently

ORGS coating on carbon steel after many surface treatments has been studied electrochemically and gravimetrically in 3% NaCl aqueous solutions to understand the corrosion prevention mechanism better.

#### 3.3.1 Gravimetric analysis

During the 30 day experiment, it was monitored how much weight was lost on carbon steel and shielded carbon steel surfaces at 30 °C. It is possible to quantify the corrosion rate (*υ*_corr_) of steel in millimeters per year (mpy) on both uncoated and coated specimens.^10,11^7
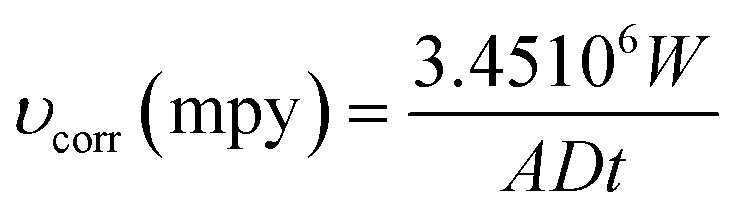
where *W* indicates weight loss (g), *D* represents the specimen density (7.85 g cm^3^), *A* represents coupon area (cm^2^), and *t* represents the exposure duration (h). Mass loss from coated *vs.* untreated steel *W*_0_ is a straightforward way to calculate protection effectiveness:^25,26^8
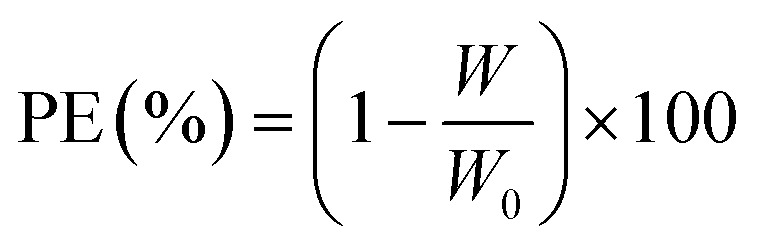


For coated and uncoated carbon steel, the gravimetric findings of pretreatment in 3% NaCl with various pretreatments are presented in Table 2: Interfaces that have been pretreated with argon or coated provide superior corrosion resistance. After 30 days of corrosion in 3%, NaCl solutions on argon pretreated samples, protection effectiveness of 81% was found. To evaluate the corrosion behavior of carbon steel interfaces after various pretreatments, coated and uncoated carbon steel interfaces were submerged in a 3% aqueous NaCl solution for up to one month. Silicon-based coatings on carbon steel are believed to help protect metals from corrosion due to their enhanced hydrophobicity, which repels water from the steel/ORGS layer. Additionally, the coating's adhesion stability is intended to be satisfactory in aqueous solutions for an extended time.

**Table 2:** Study findings on mass loss

Interface	Duration of submersion (days)	Weight loss (g × 10^−3^)	*υ*_corr_ (mpy)	PE (%)
Carbon steel	7	3.04 ± 0.03	1.51	0
	15	5.13 ± 0.04	1.19	0
	30	28.71 ± 0.04	3.33	0
Coated steel – no pre-treatment	7	0.95 ± 0.03	0.47	69
	15	2.66 ± 0.03	0.61	48
	30	19.01 ± 0.03	2.20	34
Coated steel – nitrogen pre-treatment	7	0.27 ± 0.03	0.18	91
	15	0.21 ± 0.03	0.48	59
	30	20.72 ± 0.03	2.40	28
Coated steel – argon pre-treatment	7	0.19 ± 0.03	0.09	99
	15	0.38 ± 0.03	0.08	93
	30	0.55 ± 0.03	0.63	81

**Fig. 4:**
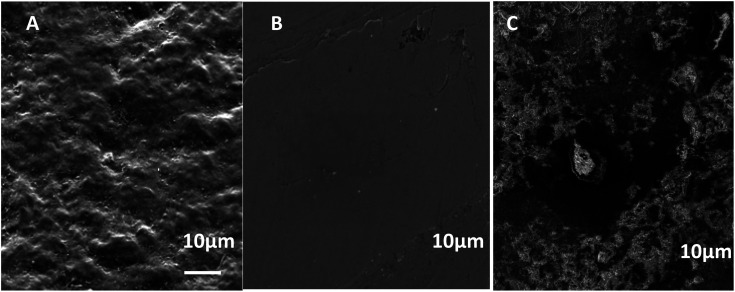
SEM images of (A) XC38 polished, (B) N_2_ pretreat. (C) Ar pretreat after immersion in a 3% NaCl aqueous solution for one month.

Indeed, it is well established that one of the critical factors affecting corrosion stability is the adhesion of metal/ORGS film interfaces, which is influenced by the quantity of water present, the temperature, the build-up of mechanical stress, and the existence of flaws and pores. To determine the efficiency of the ORGS film in protecting carbon steel against corrosion and its adherence to the metal, SEM images were taken after one month of immersion in 3% NaCl aqueous solutions (Fig. 4). In the case of unprotected carbon steel, the production of corrosion products in the form of rust is readily visible in the SEM picture (Fig. 4B). The identical phenomenon was found on a carbon steel surface pretreated with N_2_. As a result, this ORGS film is unsuitable for protecting the underlying metal contact. In the case of Ar pretreatment, no apparent corrosion products are seen (Fig. 4C), confirming the polymer's firm adherence to the polished steel surface and its corrosion-protective character.

#### 3.3.2 Measurements of the open circuit potential (*E*_OCP_)

**Fig. 5:**
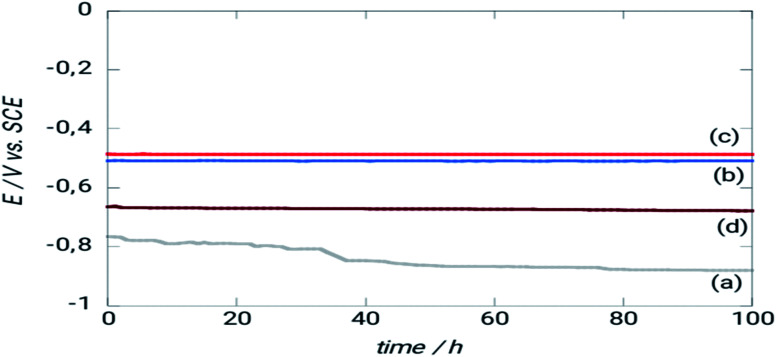
Time-dependent evolution in a 3% NaCl solution of the abundant potential of (a) XC38 for different surface pretreatments (b) without pretreatment; (c) pretreated with N_2_; (d) pretreated with Ar.

A metal interface's corrosion activity may be determined by monitoring the open circuit potential (*E*_OCP_) over time.^27,28^ The *E*_OCP_ evolution is shown in Fig. 5 when the materials are submerged in the salt solution (NaCl 3%) for uncoated and coated carbon steel.

After roughly 40 hours of immersion in NaCl 3%, a drop in *E*_OCP_ is seen due to iron dissolution.^29^ The steel covered with ORGS films had a different behavior from that of the uncoated steel. Because the *E*_OCP_ was shifted to more positive potential values, the interfaces showed increased corrosion resistance, and there was no noticeable change in *E*_OCP_ throughout the tests.

#### 3.3.3 Measurements of quasi-steady-state linear polarization

**Fig. 6:**
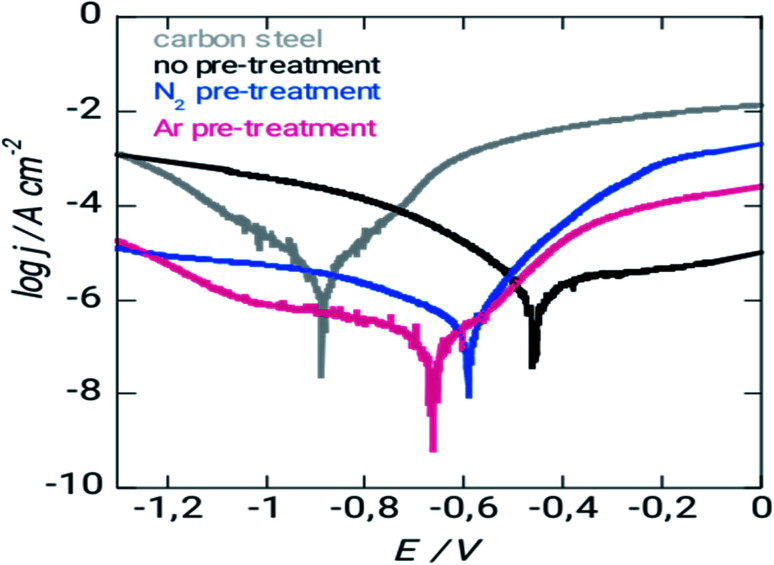
Polarization curves of XC38 for various treatment methods in 3% NaCl of solution during seven days.

Fig. 6 illustrates how pretreatment influences surface corrosion potentials using potentiodynamic polarization curves.

Corrosion protection properties of silicon coatings Fig. 5 shows the potentiodynamic polarization curves of uncoated and coated steel with different pretreatment. The corrosion current density (*i*_corr_) decreased, and the corrosion potential (*E*_corr_) increased (positive shift) for steel with argon and nitrogen pretreatment. The coated surface has a protection efficiency of 96% and 93% coated steel – argon pretreatment and coated steel – nitrogen pretreatment, respectively (Table 3). Overall, these results indicate adequate corrosion protection on the surface of the steel and more marks for argon pretreatment in corrosive environments. This coating may be helpful for potential applications that require chemically stable deposits. Eqn (8) can use the corrosion current densities *j*_corr_ and *j*_0corr_ for coated and uncoated steel to determine the corrosion protection (CP) coating.^30,31^ As with the mass loss studies, the data show that the argon-treated interface has the greatest corrosion resistance.

**Table 3:** Parameters derived electrochemically from potentiodynamic polarization curves

Interface	The duration in days of submersion	*E*_corr_ (V)	*j*_corr_ (A cm^−2^)	PE (%)
Carbon steel	7	−0.88	7.2 × 10^−6^	0
	15	−0.88	3.5 × 10^−5^	0
	30	−0.88	1.1 × 10^−4^	0
Coated steel – no pre-treatment	7	−0.46	2.5 × 10^−6^	65
	15	−0.46	3.6 × 10^−6^	50
	30	−0.48	4.7 × 10^−6^	37
Coated steel – nitrogen pre-treatment	7	−0.58	4.5 × 10^−7^	93
	15	−0.59	2.9 × 10^−6^	59
	30	−0.59	5.3 × 10^−6^	26
Coated steel – argon pre-treatment	7	−0.67	2.7 × 10^−7^	96
	15	−0.67	5.3 × 10^−7^	92
	30	−0.67	1.3 × 10^−6^	82

#### 3.3.4 Electrochemical impedance spectroscopy (EIS)

**Fig. 7:**
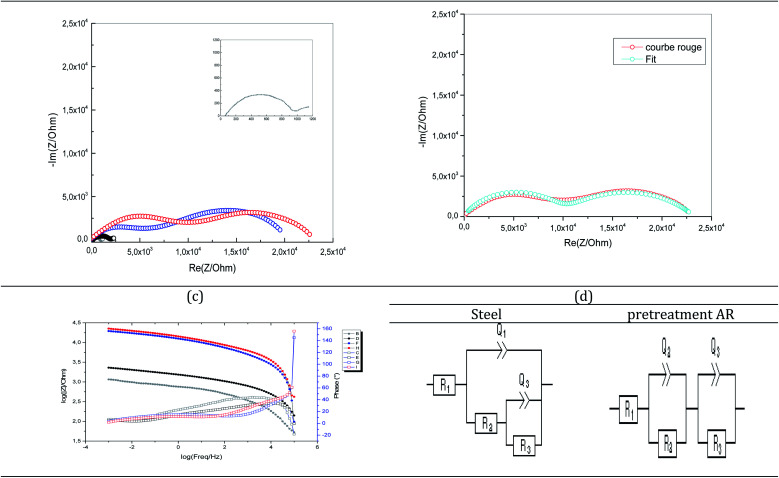
(a) Experimental and (b) fitting EIS (Nyquist) of XC38 (inset, grey) 2.5 μm ORGS coated (black: without pretreatment; blue: pretreated with N_2_; red: pretreated with Ar), (c) experimental EIS (BODE) and (d) equivalent circuit proposed of XC38 (steel, and pretreated with Ar).

An open-circuit potential amplitude of 10 mV was used to conduct further electrochemical impedance experiments, along with a frequency range of 2 mHz to 100 kHz. Fig. 7A shows a Nyquist plot with ORGS polymers in 3% NaCl coating the steel. Coated steel has two material constants in the high and medium frequency bands, while carbon steel only has one.

EIS analysis was used to evaluate the corrosion resistance of coatings without affecting the performance of the layer. Thus, EIS analysis was performed on uncoated steel and various pretreated coatings. Nyquist and Bode's plots were collected for these coatings during immersion in a 3.5% NaCl solution (Fig. 7). The Nyquist plot of the carbon steel without coating consisted of one capacitive loop (Fig. 7b). In contrast, the coated samples with different pretreatments indicate two loops generally included the high-frequency semicircular loop, which contained the information about the coating, and the low-frequency semicircular loop had information about the processes related to the reactions of the electrode surface.^31^ According to the literature, the low-frequency impedance modulus (*Z*|0.01 Hz) is a suitable parameter to characterize the protective properties of the coating.^32^ The Bode plot of argon pretreatment (Fig. 7c) showed that the value of |*Z*|0.01 Hz was 10^5^ Ω cm^2^. However, it has is of the order of 10^3^ Ω cm^2^ for untreated steel. This means that the protective property is lower than the coating with argon pretreatment at immersion.

The Nyquist and Bode plots of the coatings with different pretreatments were fitted *via* the equivalent circuit (Fig. 7d), where *R*_1_, *R*_2,_*R*_3_, *Q*_1_, and *Q*_3_ are the solution resistance, polarization resistance, constant phase element representing double-layer capacitance, and charge transfer resistance, respectively. The equivalent circuit was used when two capacitive loops appeared in the Nyquist plot. The green line indicates the fitted curves in Fig. 6 and corresponds well to the experimental data. Table 4 shows that the coated carbon steel interfaces have better charge transfer resistance than the uncoated carbon steel interfaces. The untreated coated carbon steel measured 341.8 Ω cm^2^, while the nitrogen and argon plasma-treated surfaces recorded 9.150 and 9.881 Ω cm^2^. The charge transfer of a coated steel specimen after immersion in a 3% NaCl solution is shown in Fig. 7.

**Table 4:** Impedance parameters and corrosion resistance of the steel and coated steel with different pretreatment

	Inset	Without pretreatment	Pretreated with N_2_	Pretreated with Ar
*R*_1_ (Ω)	47.59	115.6	22.68	103.8
*Q*_2_ (F s^(*a* − 1)^)	5.29 × 10^−6^	1.49 × 10^−6^	2.51 × 10^−6^	2.81 × 10^−7^
*a*_2_	0.7836	0.6573	0.4742	0.6427
*R*_2_ (Ω)	**341.8**	**1824**	**9150**	**9881**
*Q*_3_ (F s^(*a* − 1)^)	2.74 × 10^−8^	0.02 635	2.36 × 10^−5^	5.23 × 10^−5^
*a*_3_	0.4882	0.5017	0.6707	0.5267
*R*_3_ (Ω)	580	197	11 180	13 298
*s*_3_ (Ω s^−1/2^)	17.38			

The steel sample exhibited a reduced initial charge transfer resistance, which increased throughout the 100 hour immersion.

### 3.4. Theoretical study

Frontier molecular orbitals include the highest occupied (HOMO) and lowest unoccupied (LUMO) molecular orbitals. Single-electron excitation of HOMO to LUMO describes the transition from the ground state to the first excited state, known as electronic absorption. As the HOMO–LUMO gap widens, so does the system's kinetic stability. Electron transport from the ground state HOMO to the excited state LUMO needs more energy. Consequently, the HOMO–LUMO gap and other chemical descriptors (*e.g.*, chemical potential (*μ*), global hardness (*η*), and electrophilicity index (*ω*)) are provided in Table 5 for ORGS and Fe_18_ cluster pure components.

**Table 5:** DFT global reactivity descriptors

	*E*_HOMO_ (eV)	*E*_LUMO_ (eV)	GAP (eV)	*χ*	*η*	*Ω*	Δ*N*
ORGS	−6.401	0.128	6.529	3.137	3.265	1.507	0.251
Fe_18_	−3.868	−3.691	0.177	3.780	0.089	80.650	—

The ORGS molecule is the most stable because it has the most significant HOMO–LUMO gap and hardness, measuring 6.529 and 3.265 eV (Table 5). Due to its low gap and hardness, the Fe_18_ cluster was the most reactive material. The donor/acceptor ability could be calculated by calculating the cross-gap values:

9 GAP1 = *E*_LUMO_(Fe) − *E*_HOMO_(ORGS); GAP2 = *E*_LUMO_(ORGS) − *E*_HOMO_(Fe)

GAP1 and GAP2 calculated values were 2.71 and 3.996 eV, respectively. The lowest Gap value shows the possible way of electrons transfer, which means that ORGS is the electron donor (HOMO) and Fe_18_ is the electron acceptor (LUMO). The higher calculated global electrophilicity index (*ω*) of Fe_18_ compared with ORGS confirms the acceptor character of Fe_18_.

The positive calculated value of Δ*N* indicates a more remarkable electron-donating ability of the ORGS to the metal surface.

**Fig. 8:**
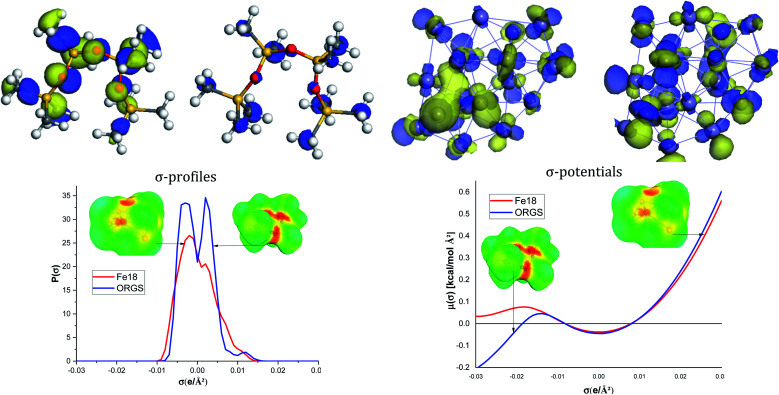
DFT global reactivity descriptors and COSMO-RS results.

As shown in Fig. 8, the ORGS the HOMO locations are located on the methyl carbons, Si, and O atoms. HOMO or LUMO iron orbitals represent the ability to behave as a donor or an electron acceptor. The ORGS and Fe_18_ binary interacting system's charge distribution may be seen on the Cosmo surfaces. The complex's non-polar regions are green, the hydrogen bond acceptor (HBA) region is red, and the hydrogen bond donor (HBD) region is blue.

The *σ*-profiles show that the two molecules are mainly non-polar except low HBA regions characteristic of the oxygen atoms of the ORGS and some iron atoms of the Fe_18_ cluster. *σ*-Potentials show that the interaction between ORGS and Fe_18_ clusters is not possible in the polar region (HBA or HBD) because Fe_18_ can't make H-bonding (*μ*(*σ*) > 0). The only possible interaction could be done in the non-polar region where *μ*(*σ*) < 0 of the Fe_18_ cluster.

**Fig. 9:**
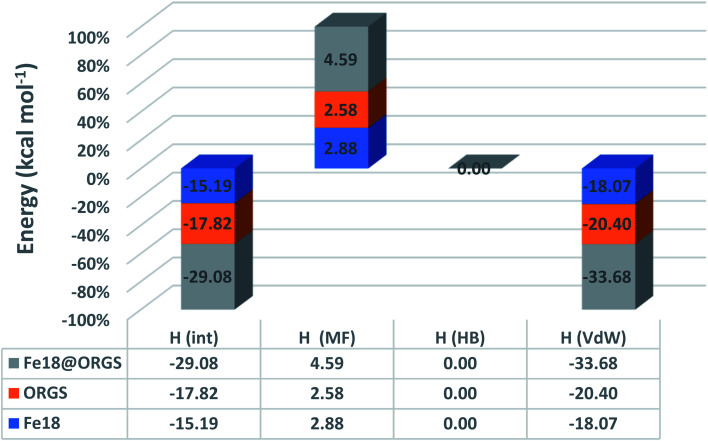
ORGS@Fe_18_ interaction energies.

Fig. 9 demonstrates that the van der Waals type *H*(vdW) = −20.40 and −18.07 kcal mol^−1^ for ORGS and Fe_18_ is the major contributor to the interaction energy in mixing the two molecules. *H*(MS) enthalpy electrostatic contributions were found to be less critical. Precisely as was expected, there was no hydrogen bonding (HB). For the ORGS, −17.82 kcal mol^−1^ was the maximum mixing enthalpy, *H*(int).

**Fig. 10:**
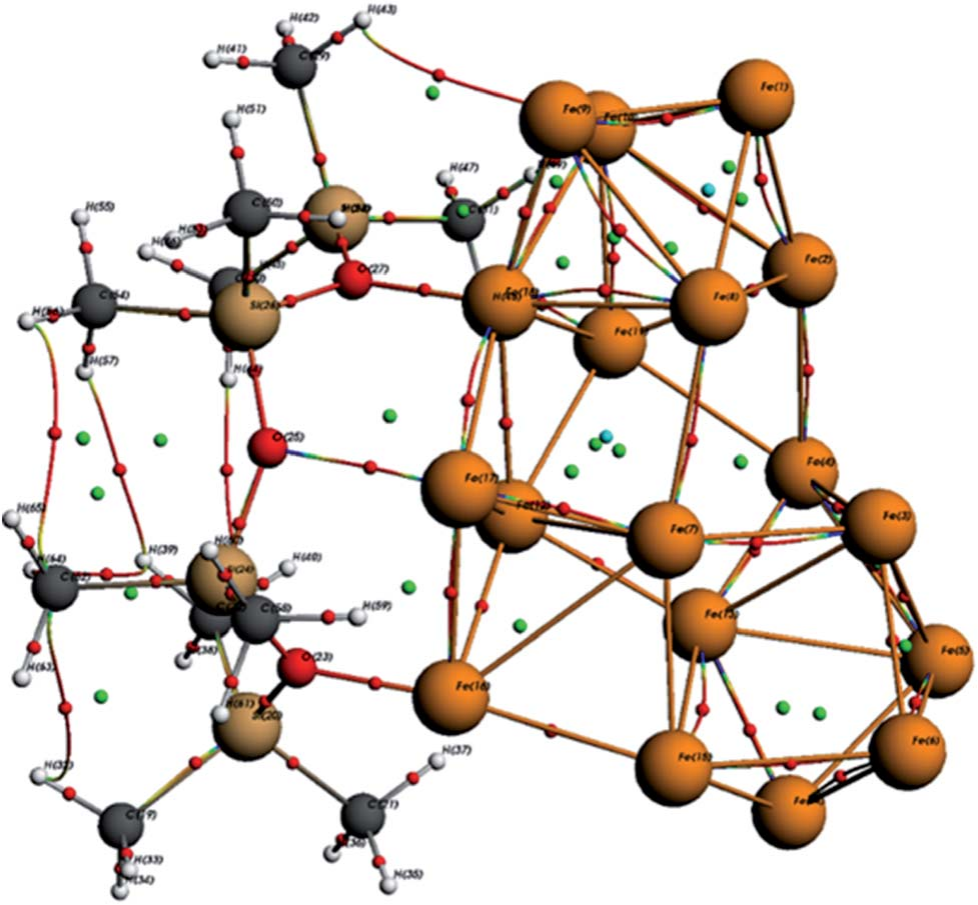
ORGS@Fe_18_ AIM analysis.

As seen in Fig. 10, the optimized ORGS/Fe_18_ complex exhibits a molecular graph, and the topological properties of the interaction contacts at the BCP are listed in Table 6.

**Table 6:** BCP interaction contact topological characteristics in the ORGS@Fe_18_ complex

BCP	*ρ*(*r*) (Ha)	∇^2^*ρ*(*r*) (Ha)	*G*(*r*) (Ha)	*V*(*r*) (Ha)	*E*_HB_ (eV)	*H*_C_ (Ha) = *G* + *V*	*G*/|*V*|
75	1.62 × 10^−2^	2.68 × 10^−2^	7.46 × 10^−3^	−8.21 × 10^−3^	−1.12 × 10^−1^	−7.47 × 10^−4^	9.09 × 10^−1^
95	1.35 × 10^−1^	8.36 × 10^−1^	2.42 × 10^−1^	−2.74 × ^−1^	−3.73	−3.27 × 10^−2^	8.81 × 10^−1^
111	1.79 × 10^−2^	1.56 × 10^−1^	2.95 × 10^−2^	−2.01 × 10^−2^	−2.73 × 10^−1^	9.48 × 10^−3^	1.47
118	2.44 × 10^−2^	1.66 × 10^−1^	3.36 × 10^−2^	−2.56 × 10^−2^	−3.49 × 10^−1^	7.98 × 10^−3^	1.31
168	1.59 × 10^−1^	4.61 × 10^−1^	2.11 × 10^−1^	−3.08 × 10^−1^	−4.19	−9.62 × 10^−2^	6.87 × 10^−1^
173	7.27 × 10^−2^	6.92 × 10^−1^	1.52 × 10^−1^	−1.30 × 10^−1^	−1.77	2.13 × 10^−2^	1.16

In BCPs, the *ρ*(*r*) values range from [0.0162, 0.159] Ha and ∇^2^*ρ*(*r*) is positive, indicating the existence of Hydrogen bonds interaction in the case of O⋯Fe and H⋯Fe, respectively (see Fig. 9). A summary of the findings may be found in the ESI.†

For BCPs 111, 118, and 173, positive ∇^2^*ρ*(*r*) and *H*_C_ values imply a weak molecular bonding (electrostatic interaction) between the atoms of the compounds (see Fig. 9). In this case *G*/|*V*| > 1, the ability to group electrons, *V*(*r*) is less than the ability to dilute them, *G*(*r*). *E*_HB_ < 0 characterizes the poor hydrogen bonding.^33^ The bonds O (25)⋯Fe (17), H (48)⋯Fe (11) and O (23)⋯Fe (16) are all determined to be of a physical character (no covalent bonding). At the BCPs 75, 95, and 168, the negative *H*_C_ and positive ∇^2^*ρ*(*r*) values in the case of H (43)⋯Fe (10), 0 (27)⋯Fe (18) and H (49)⋯Fe (10) demonstrates the partially covalent character. This result is corroborated by the fact that the ability to group electrons, *V*(*r*), is stronger than the ability to dilute them through electronic mobility, *G*(*r*) where 
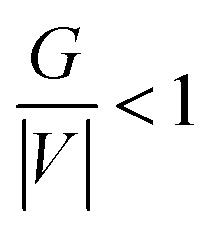
.

## 4. Conclusion

The present study demonstrates the successful deposition of thin SiO_*x*_-like coatings onto carbon steel using plasma-assisted chemical vapor deposition. The concept is straightforward to apply to massive surfaces. The created architecture was hydrophobic and adhered to the underlying steel contact. A 2.5 μm thick argon pretreatment coating demonstrated exceptional adhesion stability and hydrophobicity (water contact angle of 117 ± 2°). In a 3% NaCl aqueous solution, the inhibition efficacy of the silica films against corrosion was determined. While shallow SiO_*x*_-like coating layers (2.5 μm) without pretreatment do not protect carbon steel surfaces from corrosion, larger silica layers with argon pretreatment do. Indeed, polarization investigations on an argon pretreatment steel surface revealed a protection efficiency of 96%. This is somewhat less than the 99% protection efficiency observed by Pech *et al.*^8^ and Delimi *et al.*^34^ on steel samples covered by a 600 nm thick silica-based coating.^3,34^ However, the findings indicate that three times thinner amorphous silica films already possess a significant level of protection. Thus, thin SiO_*x*_-like layers constitute an intriguing possibility for easily and affordably increasing the anticorrosion performance of diverse materials. The coatings adhered effectively to the carbon-steel surface according to the theoretical study. The van der Waals forces were revealed to account for the bulk of the interaction energy. Electrostatic forces were discovered to have a lesser effect.

## Author contributions

Amel Delimi, Hana Ferkous: experimental investigation, discussion of results, draft preparation. Souad Djellali, Amel Seddik, Kahlouche Abdessalem, Chérifa Boulechfar, Amina Belakhdar: discussion of results, draft preparation. Marina M. S. Cabral-Pinto, Manawwer Alam: theoretical investigation, discussion of results. Krishna Kumar Yadav, Byong-Hun Jeon: editing and writing, funding research. Yacine Benguerba: theoretical investigation, discussion of results, editing and writing.

## Conflicts of interest

There are no conflicts to declare.

## Supplementary Material

RA-012-D1RA08848C-s001
