# Supplementary material for: Corrosion protection performance of silicon-based coatings on carbon steel in NaCl solution: a theoretical and experimental assessment of the effect of plasma-enhanced chemical vapor deposition pretreatment
Source: RSC Adv. 2022 May 25;12(24):15601–12. doi: 10.1039/d1ra08848c (PMC9131146; doi:10.1039/d1ra08848c)
Supplement: RA-012-D1RA08848C-s001 [file RA-012-D1RA08848C-s001.pdf]

Electronic Supplementary Material (ESI) for RSC Advances.
This journal is © The Royal Society of Chemistry 2022

Corrosion Protection Performance of silicon-based coatings on carbon Steel in NaCl Solution: A theoretical and experimental assessment of the effect of plasma-enhanced chemical vapor deposition pretreatment

Amel Delimi[1], Hana Ferkous[2], Manawwer Alam[3], Souad Djellali[4] , Amel Seddik[5,6], Kahlouche Abdessalem[7], Chérifa Boulechfar[1], Amina Belakhdar[8], Krishna Kumar Yadav[9], Marina M.S. Cabral-Pinto[10], Byong-Hun Jeon[11], Yacine Benguerba[12]*

[1] *Laboratoire de Génie méncanique et Matériaux, Faculty of Technology, University of Skikda, 21000,* ***Algeria***

[2] *Département of Technology, University of Skikda, 21000, Skikda,* ***Algeria***

[3]*Department of Chemistry, College of Science, King Saud University, P.O. Box 2455, Riyadh, 11451, Saudi Arabia*

[4] *Laboratoire de Physico-Chimie des Hauts Polymères (LPCHP)", Faculty of Technology, University Ferhat Abbas Setif1, 19000, Setif,* ***Algeria***

[5] *Scientific and Technical Research Center in Physico-chemical Analysis, BP 384, Bou-Ismail industrial zone, RP 42004,* ***Tipaza*****,** ***Algeria***

[6] *Nanomaterials, corrosion and surface treatment laboratory (LNMCT), BP 12, Badji Mokhtar University, 23000 Annaba* ***Algeria***

[7] *Centre de recherche et technologie industrielle" CRTI Cheraga* ***Algeria***

[8] *Laboratoire Matériaux et Systèmes Electroniques", University of Bordj Bou Arreridj, 34000,* ***Algeria***

[9] *Faculty of Science and Technology, Madhyanchal Professional University, Ratibad, Bhopal 462044,* ***India***

[10] *Geobiotec Research Centre, Department of Geoscience, University of Aveiro, 3810-193, Aveiro,* ***Portugal***

[11] *Department of Earth Resources and Environmental Engineering, Hanyang University, Seoul 04763,* ***Republic of Korea***

[12] *Department of process engineering, Faculty of technology, Ferhat Abbas Setif1 University, Setif,* ***Algeria***

***Corresponding Author**: yacinebenguerba@univ-setif.dz

Supplementary data

ATOMS	#BP	BCP	Atom	Atom
1 Fe	1	74	1	2
2 Fe	2	70	1	9
3 Fe	3	79	2	4
4 Fe	4	78	2	8
5 Fe	5	81	2	10
6 Fe	6	104	3	4

7 Fe	7	112	3	5
8 Fe	8	113	3	6
9 Fe	9	102	3	7
10 Fe	10	92	4	5
11 Fe	11	128	4	13
12 Fe	12	127	5	6
13 Fe	13	137	6	14
14 Fe	14	130	6	15
15 Fe	15	82	7	8
16 Fe	16	108	7	17
17 Fe	17	72	8	9
18 Fe	18	80	8	18
19 C	19	68	9	10
20 Si	20	76	9	18
21 C	21	101	10	11
22 C	22	69	10	18
23 O	23	75	10	43
24 Si	24	168	10	49
25 O	25	118	11	48
26 Si	26	136	12	13
27 O	27	139	12	16
28 Si	28	105	12	18
29 C	29	148	13	14
30 C	30	140	13	15
31 C	31	132	15	16
32 H	32	96	16	17
33 H	33	173	16	23
34 H	34	100	17	18
35 H	35	111	17	25
36 H	36	95	18	27
37 H	37	156	19	20
38 H	38	160	19	32
39 H	39	161	19	33
40 H	40	163	19	34
41 H	41	159	20	21
42 H	42	158	20	22
43 H	43	174	20	23
44 H	44	177	21	35
45 H	45	162	21	36
46 H	46	176	21	37
47 H	47	175	22	38
48 H	48	155	22	39
49 H	49	157	22	40
50 C	50	114	22	44
51 H	51	147	23	24
52 H	52	133	24	25
53 H	53	131	24	58
54 C	54	145	24	62
55 H	55	115	25	26
56 H	56	117	26	27
57 H	57	93	26	50
58 C	58	123	26	54
59 H	59	170	27	28
60 H	60	120	28	29
61 H	61	110	28	30
62 C	62	106	28	31
63 H	63	90	29	41
64 H	64	169	29	42
65 H	65	167	29	43
	66	143	30	44
	67	146	30	45
	68	138	30	46
	69	178	31	47

	70	129	31	48
	71	107	31	49
	72	154	32	62
	73	89	39	57
	74	151	39	64
	75	84	50	51
	76	166	50	52
	77	83	50	53
	78	119	54	55
	79	172	54	56
	80	135	54	57
	81	109	56	62
	82	121	58	59
	83	116	58	60
	84	134	58	61
	85	153	62	63
	86	152	62	64
	87	142	62	65
